# Sib-recruitment for studying migration and its impact on obesity and diabetes

**DOI:** 10.1186/1742-7622-3-2

**Published:** 2006-03-13

**Authors:** Tanica Lyngdoh, Sanjay Kinra, Yoav Ben Shlomo, Srinath Reddy, Dorairaj Prabhakaran, George Davey Smith, Shah Ebrahim, the Indian migration study group

**Affiliations:** 1 Centre for Chronic Disease Control, T-7, Green Park Extn, Delhi, 110 016, India; 2 Department of Social Medicine, University of Bristol, Senate House, Tyndall Avenue, Bristol, UK; 3 All India Institute of Medical Sciences, Angari Nagar, Delhi, India; 4 Department of Epidemiology & Population Health, Keppel St, London School of Hygiene & Tropical Medicine, London, UK

## Abstract

**Background:**

Urban-rural comparisons are of limited relevance in examining the effects of urban migration in developing countries where urbanisation is due to growth of existing urban populations, expansion of urban boundaries, and rural in-migration. Cultural, genetic and life-style backgrounds of migrants and host populations further limit the value of rural-urban comparisons. Therefore we evaluated a sib-comparison design intended to overcome the limitations of urban-rural comparisons.

**Methods:**

Using the framework of a current cardiovascular risk factor screening study conducted in Indian factories, we recruited the non-migrant rural sibs of migrant urban factory workers and the urban sibs of non-migrant factory workers. The response rate, completed interviews and examinations conducted were assessed. Adequacy of generic food frequency questionnaires and WHO quality of life questionnaire were assessed.

**Results:**

All the urban factory workers and spouses approached agreed to be interviewed. Of the 697 participants interviewed, 293 (42%) had at least one rural dwelling sibling. Twenty (22%) siblings lived further than 100 km from the study site. An additional 21 urban siblings of non-migrant factory workers were also investigated to test the logistics of this element of the study. Obesity (BMI >25 kg/m2) was more common in rural sibs than urban factory workers (age adjusted prevalence: 21.1% (17.1 to 25.0) vs. 16.1% (11.9, 20.3). Diabetes prevalence (fasting plasma glucose greater than 126 mg/dl) was higher than expected (age-adjusted prevalence: 12.5% (22 out of 93) in urban migrants and 4.5% (8 out of 90) in rural non-migrant sibs.

**Conclusion:**

The sib-comparison design is robust and has been adopted in the main study. It is possible that simple urban-rural study designs under-estimate the true differences in diabetes risk between migrants and non-migrants.

## Background

The health consequences of migration are classically conceptualised in terms of exposures experienced in the home country; exposures acquired in the new country; health or disease selection of those who migrate; and, exposures due to the process of migration itself [1]. The usual typologies of migrant – settler, contract worker, student, professional, illegal immigrant and refugee – are likely to be of less value in understanding health consequences than the process of migration itself, which is complex. Age at migration and gender are likely to be key factors [2], but the speed, reasons for migration, together with the social disruption involved, might be expected to influence the health consequences experienced. For example, case studies of forced migration in Cambodia and Zaire have highlighted the increased mortality risk experienced by all migrant types [3].

Geographic differences in disease risk are common but ecological studies correlating national disease risk with levels of possible risk factors can only provide weak evidence to test causal hypotheses as association at the population level may not reflect an association at the individual level. Migrating populations can provide stronger evidence as people migrating from low to high risk populations would be expected to acquire the high risk of the host population if the disease is largely environmentally determined. In a classic migrant study of Tokelau islanders following a hurricane in 1966, migrants to New Zealand had a higher risk of diabetes and were more obese compared to non-migrants, which was related to their duration of stay and family size, but not age at migration [4,5]. These findings suggest a rapid acquisition of health behaviours increasing the risk of diabetes. By contrast, the migration experience of Japanese migrating to the USA has been more gradual, they have joined kith and kin, and the opportunity to maintain a Japanese lifestyle has existed. Despite this lack of apparent acculturation, the risk of diabetes amongst Japanese living in Hawaii and Los Angeles is 2–3 times higher than in Japanese living in Japan [6]. Insulin levels are higher in those living in the USA than Japan for the same degree of obesity and glucose intolerance, suggestive of acquired insulin resistance. Importantly, those who retain a traditional lifestyle and are not obese are less likely to be diabetic, although the risk is still higher than in Japan [7]. A rise in obesity does not appear to be an inevitable consequence of migration; studies have found declines in obesity with increased acculturation following migration [8,9]. The effects of the migration process itself have been explored in only a very limited way so far [10].

Urban-rural comparisons are of limited relevance in examining the effects of urban migration in developing countries as, unlike Europe [11], the urbanisation of developing countries is due to growth of existing urban populations, expansion of urban boundaries, and rural to urban migration [12]. Cultural, genetic and life-style backgrounds of migrants and host populations further limit the value of rural-urban comparisons. Furthermore, such comparisons do not examine the interaction of migration with diabetes risk, such that those who migrate may be at higher or lower risk than the indigenous urban population. Therefore we evaluated a little-used sib-comparison study design to overcome the limitations of urban-rural comparisons in examining effects of migration.

## Methods

### Study design

The study was nested within a Cardiovascular Disease Risk Factor Study (CVDRFS) in four Indian cities (Bangalore, Lucknow, Nagpur, and Hyderabad), situated geographically in the north, centre and south of the country, and covering sites where rural-urban migration occurs. For the preliminary work reported here, work was confined to two of the sites, Lucknow and Hyderabad. Participants in the CVDRFS baseline survey, together with their co-resident spouses, were asked about rural-to-urban migration and those responding positively, together with a 25% random sample of non-migrants, were invited to participate in the study. Indian Census 2001 definitions were used to classify areas as urban or rural based on population size and density and non-agricultural employment [13]. Migration status was attributed only to intra-generation migrants (i.e. 'first-generation') and of at least one year's duration. Place of origin was identified using a commercial GIS application of the Indian census produced for the study. The software enabled village level unique census identifier codes to be assigned to each participant's place of origin, permitting electronic linkage to relevant census data.

**Figure 1:**
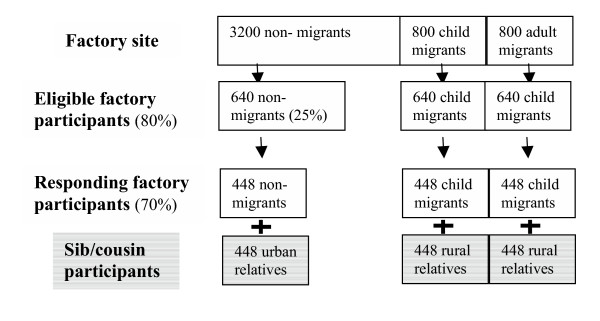
Sampling strategy for each factory.

Each participant was asked to identify one non-migrant full sibling of the same sex and closest to them in age. In the case of migrants whose siblings had also migrated, a half-sib, and if not available, then the closest cousin and still resident in the village of origin was recruited instead of the full sibling. For non-migrant workers, siblings who resided in the same city but did not work in the factory were recruited to enable prevalence of obesity and diabetes among factory workers and their sibs to be compared, estimating any healthy worker effect and more generalisable urban prevalence rates than that obtained solely from factory workers. The sampling strategy is shown in Figure 1.

#### Main outcome measures

The main outcomes under study are obesity and diabetes. Overweight and obesity were defined using Indian reference ranges as a body mass index (BMI) greater than 23 kg/m^2 ^and obesity 25 kg/m^2 ^[14]. Measures of central adiposity (waist-hip ratio) were made and is planned in the main study to assess body fat composition using skin-fold measures at sub-scapular, triceps, and medial calf sites and use predictive equations derived from Indian populations validated using hydrodensitometry [15]. Diagnosis of diabetes was made using both the new (fasting plasma glucose greater than 7.0 mmol/l) and old (fasting plasma glucose greater than 7.8 mmol/l) WHO criteria, in which a glucose tolerance test is not required [16].

#### Assessment of dietary intake and physical activity

Diet was assessed by interviewer-administered semi-quantitative food frequency questionnaire using validated and reproducible food frequency methodology [17], that has been widely used in India [18,19]. Detailed nutrient values for Indian foods were used to calculate macro and micro nutrients [20].

Physical activity status was assessed by interviewer-administered questionnaire. No validated Indian physical activity questionnaire exists, but the energy cost of a range of activities has been assessed, enabling METS enabling metabolic equivalents of task (METs, 1 MET is equivalent to a metabolic rate consuming 1 kilocalorie per kilogram of body weight per hour) to be derived from a 24-hour activity record [21,22]. Previous studies have used simple short questions about occupation, leisure time activity and household work, and related those to outcomes of interest such as obesity and related disorders [23,24].

The WHO-QoL [25], a generic measure of health-related quality of life, was administered to examine the healthy worker effect, potential health benefits of migrant status, and the psycho-social consequences of obesity and diabetes.

#### Laboratory assays

Blood samples were transported weekly to All India Institute of Medical Sciences (AIIMS), Delhi, having been separated and stored at -20°C locally. Serum HDL-cholesterol was estimated directly by an elimination method [26], total cholesterol was estimated by an enzymatic endpoint method, triglycerides by GPO-PAP method, and glucose by GOD-PAP method using RANDOX kits [27]. For quality assurance the Cardiac Biochemistry Lab, AIIMS, is part of the UK National External Quality Assessment [28]

## Results

All the urban factory workers and spouses approached agreed to be interviewed. Of the 697 participants interviewed during the pilot phase of the work, 293 (42%) had at least one rural dwelling sibling. Of these, 155 (53%) participants also indicated willingness to invite their rural siblings for clinical examination. At the close of the pilot, clinical examination had been completed on 92 of the 215 (43%) rural siblings invited to participate. Completeness of data recording was good with very few missing values for key variables (See Table 1). During the first 6 months of the main study, of 5663 people assessed for inclusion in the study, only 338 (6.8%) refused to take part. Among the 2918 eligible and willing to take part, 1994 (68%) were rural-urban migrants. Of these, 1315 (45%) had a rural-dwelling sib who was willing to attend, 209 (16%) of whom have already attended for interview and clinical examination.

**Table 1:** Distributions of major variables collected, and amount of missing data

Variable	Mean (SD), or percent, total number of participants in brackets	Number (%) missing or invalid
Percent current smokers	22.8% [189]	-
Percent current alcohol users	10.6% [189]	-
Body Mass Index (kg/m^2^)	23.5(4.3) [188]	1(0.5%)
Blood cholesterol (mg/dl)	178 (39.3) [181]	8 (4.2%)
Blood glucose (mg/dl)	103.2 (36.6) [181]	8 (4.2%)
Blood pressure-Systolic (mmHg)	128.8 (18.9) [187]	2 (1.1%)
Blood pressure-Diastolic (mmHg)	80.9 (11.2) [187]	2 (1.1%)

Identifying participants' place of origin and the location of sibs was facilitated by using an electronic version of the Indian census. Places with the same name could be verified in real time and linked to local geographic features such as railway stations and larger towns, and assigned their correct Indian census identification code number. Twenty (22%) siblings lived further than 100 km from the study site. An additional 21 urban siblings of non-migrant factory workers were also investigated to test the logistics of this element of the study.

Comparison of urban participants and rural sibs showed that urban residents were relatively older and more educated, consumed less tobacco and alcohol, but had higher age-adjusted prevalence of hypertension and diabetes (see Table 2). Contrary to expectations, age-adjusted obesity and central obesity prevalences were higher in rural sibs than in urban factory workers although estimates were imprecise owing to the small pilot sample. There was moderate to strong evidence of differences in smoking and drinking, raised total:HDL cholesterol ratio, hypertension and diabetes between rural and urban sibs Closer inspection of data revealed that obesity was similarly prevalent across age categories in rural participants but strongly age-dependent (more common in the older age groups) in the urban ones. Diabetes prevalence (fasting plasma glucose greater than 126 mg/dl) was higher in urban participants (age-adjusted prevalence: 12.5%) than in rural sibs (4.5%, p < 0.05).

**Table 2:** Distribution of risk factors by place of residence

	**Rural sibs of urban factory workers**		**Urban factory workers**	
	
	**% (Cases/Total)**	Age-adjusted prevalence (95% CI)*	**% (Cases/Total)**	**Age-adjusted prevalence (95% CI) $**
Mean age (SD)	41.7 (10.6)	NA	46.6 (7.6)	NA
Male sex	71.7 (66/92)	NA	80.4 (78/97)	NA
Illiteracy	21.7 (20/92)	11.8 (8.0 to 15.5)	6.2 (6/96)	3.1 (0.1 to 5.3) ***
Tobacco use (current)	30.4 (28/92)	14.5 (10.2 to 18.8)	15.5 (15/97)	7.6 (2.6 to 12.5)
Alcohol use (current)	14.1 (13/92)	6.7 (3.4 to 10.0)	7.2 (7/97)	1.7 (0.5 to 2.8) **
Obesity	26.1 (24/92)	21.1 (17.1 to 25.0)	44.3 (43/97)	16.1 (11.9 to 20.3)
Abdominal obesity	20.7 (19/92)	11.8 (8.0 to 15.6)	25.8 (25/97)	8.1 (5.2 to 11.0)
Dyslipidaemia	34.4 (31/90)	15.7 (11.1 to 20.2)	32.3 (30/93)	9.7 (6.7 to 12.7) *
Hypertension	15.4 (14/91)	9.1 (5.7 to 12.6)	39.6 (38/96)	15.2 (10.9 to 9.5)*
Diabetes	8.9 (8/90)	4.5 (1.6 to 7.4)	23.7 (22/93)	12.5 (6.7 to 18.4)*

$ Age-adjusted prevalence (95% confidence intervals) calculated by the direct method of standardisation, using the reference World population (ref: dos Santos Silva, I. Cancer epidemiology: principles and methods. Lyon: IARC, 1999.)

p-values for difference in age-adjusted prevalence rates: *** p < 0.001, ** p < 0.01, * p < 0.05

Outcome definitions:

• Current tobacco use – Any tobacco product used on a daily basis, anytime in the last 6 months

• Current alcohol use – Alcohol consumed at least 10 days/month, anytime in the last 6 months

• Obesity – body mass index greater than 25 kg/m2

• Abdominal obesity – waist circumference greater than 88 cm (women) or 94 cm (men)

• Dyslipidaemia – total cholesterol-HDL cholesterol ratio greater than 4.5

• Hypertension – systolic blood pressure greater than 140 mmHg or diastolic blood pressure greater than 90 mmHg

• Diabetes – fasting blood sugar greater than 126 mg/dl

The simple generic food frequency questionnaire proved inadequate to capture the complexity of dietary differences between rural and urban participants and between different areas of India, and was abandoned. Consequently, 24-hour dietary recalls were conducted on urban and rural participants (n = 100 in each site) from Bangalore and Hyderabad (south India) and Lucknow (north India) and Nagpur (Central India), and the data collected are being used to produce an appropriate food frequency questionnaire.

The WHO-QoL questionnaire was found to be too time consuming to complete easily. Despite in-depth training, interviewers and participants found the questionnaire difficult as it tended to provoke an involved question and answer session. Its use was abandoned.

## Discussion

We have implemented a sib-comparison design to examine the effects of migration by using a counter-factual approach of comparing the health outcomes of migrating people with their non-migrating sibs who remain resident in the place of origin of both. This method overcomes the inherent problems of conventional rural-urban comparisons but makes allowance for temporal life-style and exposure trends that will have affected both migrants and non-migrants, for example, the increased access to energy rich foods throughout India. Preliminary findings from this small study suggest that the patterns of cardiovascular risk factors are more complex than previous rural-urban comparisons would suggest. It is unwise to over-interpret these pilot findings as the sample size is small and the estimates are imprecise.

It is important to consider potential sources of bias introduced by the sib-comparison design. As the index person is identified through association with a factory, it is likely that there will be a healthy worker effect. This will be examined by recruitment of urban dwelling sibs, making comparisons of health behaviours and CVD risk factors between them and the index persons. The sibs recruited from rural areas will not be representative of the generality of rural dwelling people, but as our purpose is not to make inferences about the rural-urban differences in behaviour and risk this is not relevant. However, it is quite likely that rural dwelling sibs who are unwell are less likely to attend for examination, resulting in attenuation of any adverse effects associated with urban migration. We will explore reasons for non-attendance to estimate the scale of this potential problem.

### Sib comparison, twin and cohort designs

The sib-comparison design was first used in a study of the effects of Irish migration to the USA in the Boston-Irish Diet Heart study. Briefly, the excess mortality among Irish-born migrants to the USA, apparent in the 1950s, prompted a study of Boston-dwelling Irish and their sibs still living in Ireland compared with Boston-dwelling Americans[29]. No differences in cardiovascular risk was found between the groups and this was probably an effect of divergent and marked secular trends in risk in Ireland (rising risk) and USA (falling risk) at the time of study [30]. We have not found any examples of this design in studies of migration in low and middle-income countries.

Although sib-comparisons appear to be like twin studies, it is important to recognize that the main purpose of twin studies is to determine the genetic contribution of diseases by examining discordance in traits between monozygotic (identical genes) and dizygotic twins (no more genetically similar than sibs). In obesity and diabetes that clearly have a genetic contribution demonstrated by heritability indicators [31], both twin and sib studies can help to tease out the environmental determinants of these health problems by controlling completely in the case of monzygotic twins, and partially in the case of dizygotic twins and sibs, for genetic variation. For example, in a comparison of identical twins discordant for exercise behaviour, there is strong evidence of an effect of exercise on body mass index, but no correlation of body mass index between twin-pairs, indicating the importance of environmental determinants of obesity [32]. Recently, it has become apparent that phenotypic differences in identical twins may be caused not only by environmental factors but also by epigenetic factors (i.e. cellular information, other than the DNA sequence, that is heritable – mechanisms include DNA methylation, genetic imprinting, and transcription regulation [33]), making their interpretation more complex [34].

Cohort studies can be used to examine migration effects but require substantial numbers of people to migrate if effects on disease events are to be studied with sufficient power. For example, in the British Regional Heart Study blood pressure and coronary heart disease were more strongly correlated with place of adult residence than place of birth, suggesting that exposures during adult life were more important determinants than genetic or early life factors [35].

### Previous studies in India

No previous study has examined the effects of rural to urban migration on obesity and diabetes in India. Comparisons of the prevalence of obesity and diabetes mellitus in rural and urban areas of India have been reported in a large, but out-dated study [36], and only one recent and reliable small-scale study [37], (see Table 3) but these do not illustrate an effect of migration per se. Peri-urban areas are reported to have an intermediate prevalence of diabetes (5.9%) and obesity (12.5%) [38]. Although these studies used different criteria from ours to define obesity and diabetes, this is unlikely to explain the marked increase in both conditions over time and the widening gradient between urban, peri-urban and rural prevalence. In a large survey of six cities, an age-adjusted diabetes prevalence of 12% was recently reported, demonstrating that rates are continuing to rise [39].

**Table 3:** Indian studies of urban-rural comparisons of obesity and diabetes prevalence

Study	Obesity prevalence (%)		Urban/Rural Ratio	Diabetes prevalence (%)		Urban/Rural Ratio
				
	Urban	Rural		Urban	Rural	
ICMR, 1975 [36]	2.5	0.6	4	1 – 3.8	0.6 – 1.9	~2
South India, 1992 [37]	22	2	11	8.3	2.4	3.5

The Indian National Family Health Survey 2 (NFHS 2) carried out in 1998–99, sampled 95,000 women of reproductive age from nationally representative areas and found a less marked urban-rural obesity ratio of 6-fold, probably reflecting the younger age of participants [40]. Diabetes prevalence was not estimated. Smaller surveys from other urban and rural regions of India report comparable prevalences of obesity and diabetes, but have not attempted to make direct comparisons using standardised methodology.).) [41-43]. Urban-rural gradients in diabetes may be higher in south than north India [36], which may reflect genetic, cultural and lifestyle differences, together with the degree of acculturation in the geographic areas compared. Indian migrants abroad appear to be at particularly high risk compared with the host populations they join. Indians living in Mauritius, Fiji, Singapore, Tanzania, Netherlands and Britain have very high diabetes prevalences (15–20%) that are much higher than in India itself and the host countries [44-46].

### Biological mechanisms

Markedly higher levels of serum insulin in urban as compared to the rural participants, including sub-samples with normal glucose tolerance, have been found [47]. This suggests that some of the effect of urbanisation may be mediated through biological factors that result in increased secretion of insulin due to tissue resistance to its actions. Although none of the Indian studies specifically studied migrants, similarly high levels of serum insulin have been reported in Asian Indians living abroad [46], in populations from other developing countries experiencing rapid urbanisation, and in migrant populations elsewhere [44,47,48]. Little is known about the ways in which migration and social patterning interact to increase this fundamental biological step in the path to development of diabetes. In the main study fasting insulin levels are being measured to provide relevant data.

Glucose intolerance, obesity, dyslipidaemia and hypertension are part of a common pathological syndrome of insulin resistance [50]. The importance of insulin resistance lies in accumulating evidence that it is causally associated with type 2 diabetes and CHD [49,51-53], and the growing view that it is the primary defect in the evolution of type 2 diabetes. Prospective studies in high-risk individuals have found that insulin resistance is the most prominent and earliest metabolic defect that can be detected in the pre-diabetic state, conferring high risk of subsequent development of diabetes [54,55]. Cytokines such as tumour necrosis factor-alpha and interleukin-6 may be the biological link between obesity and insulin resistance [56], and are raised in Indian urban slum dwellers (often recent migrants), lowest in rural villagers, with intermediate levels among the urban middle class [57]. Exploratory study of the role of cytokines as a mechanism by which migration effects operate is warranted. Understanding the ways in which variations in migration history, current environmental exposures, and life-style combine to increase risk of obesity, insulin resistance and diabetes may help us develop more effective means of controlling those aspects of migration and social patterning of life that contribute most to increasing risk.

### Implications

Several lessons have been learned in the process of conducting this extended pilot study. Although nutritional surveillance is used throughout India, and reports of validated questionnaires were found [58,59], the existing food frequency questionnaires available were not adequate for making comparisons between rural and urban areas, or for comparisons between different regions of India. This is because of marked regional differences in staple foods consumed, seasonal variability in food availability, cooking oils used and recipes used. It has proved necessary to conduct a series of in-depth studies using market place surveys to establish local availability of seasonal foods, and to produce weighed standard recipes for commonly prepared foods. From these accurate food lists have been prepared from which a food frequency questionnaire has been produced and from which dietary constituents can be determined.

We had hoped to use the WHO-QoL as this is considered to be an internationally useful and validated instrument to assess quality of life in population surveys. In practice it proved impossible to establish support in its use from the WHO nominated WHO-QoL experts, and despite training our interviewers, they experienced considerable difficulty in its use. In particular, interviewers found it hard to get beyond the initial questions (e.g. How would you rate your quality of life? How satisfied are you with your health? To what extent do you feel your life to be meaningful?) which evoked long explanations about the meaning of quality of life, sources of satisfaction in life, and the meaning of life. We have decided to drop it from the full survey. Instead we will use qualitative methods of in-depth interviewing and focus groups to explore reasons for migration and how they relate to the health of individuals and behavioural perceptions. Some focus group discussions and individual interviews with migrants have already taken place while more are ongoing. The data from these are being compiled and analysed to inform the qualitative component of the study and to develop a quantitative "westernisation" index.

Despite having adequate resources to conduct the field work, it was apparent that financial barriers for rural sibs existed and sensitive means of dealing with the wide range of social circumstances were required. We had to ensure that field staff had considerable flexibility in the allocation of costs of travel and subsistence. Furthermore, as the costs of accommodating rural sibs would fall on their urban relatives it has proved necessary to negotiate carefully who should receive reimbursements.

We correctly anticipated that attempting to record participants' place of origin would be time consuming and would yield information that would be difficult to link with village and small town unique census codes. This is because many Indian places have the same, or similar sounding names, and even with details of the state and nearest large town, errors of identification will arise. Establishing the unique identification number of the place of origin was essential as we intend to link census derived data on socio-economic status of place of origin (and if possible use data from sequential Indian censuses from 1971 to 2001), relating these data to subsequent risk of obesity and diabetes. The software was specially designed for our study but we think it will have general value in other studies, enabling the large data resources of the Indian censuses to be more widely used in epidemiologic and social research.

In conclusion, we have tested a little used means of examining rural-urban migration that appears to be of particular utility in India, avoiding the need for long-term cohort studies that would be impracticable for examining the role of migration in current burdens of obesity and diabetes.

## Competing interests

The author(s) declare that they have no competing interests.

## Authors' contributions

TL supervised field team and abstracted data, SK contributed to the conception and design of the study, trained and supervised the field team. YBS, SR, GDS contributed to the conception and design of the study. DP monitored data quality and supervised the team. SE contributed to design and coordination of the study, and drafted the manuscript. All authors read and approved the final manuscript.

**Figure 2:**
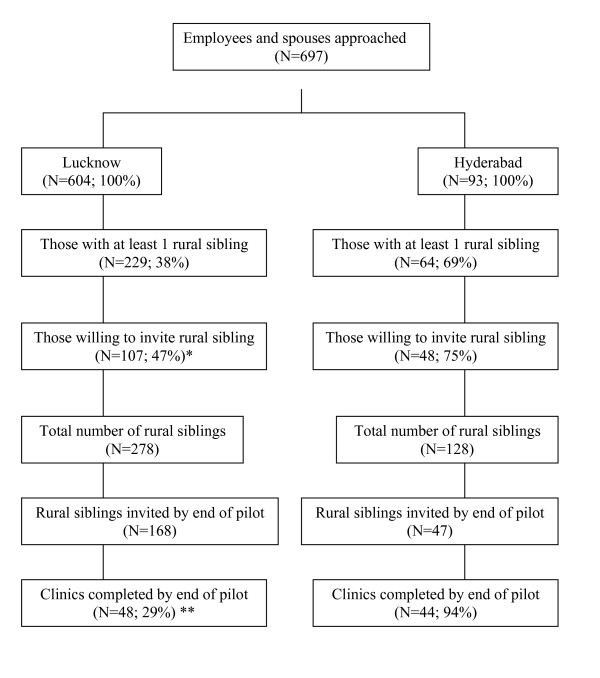
Flow chart showing recruitment of siblings. * This figure is likely to be a gross underestimation of the true figure, as the fieldwork was halted to start the main study. ** This does not represent the final response rate as the participants were requested to appear for examination anytime within a year of invitation, at a time of their convenience (such as school holidays). The pilot, however, was brought to a close after six months and this gave most siblings only 2–3 months on average to respond.
